# O.4.5-1 Device-measured physical activity and sedentary time in a national sample of Luxembourg residents: moving beyond the traditional metrics in the ORISCAV-LUX 2 study

**DOI:** 10.1093/eurpub/ckad133.209

**Published:** 2023-09-11

**Authors:** Laurent Malisoux, Anne Backes, Gloria A Aguayo, Paul James Collings

**Affiliations:** Luxembourg Institute of Health, Luxembourg; laurent.malisoux@lih.lu; Luxembourg Institute of Health, Luxembourg; laurent.malisoux@lih.lu; Luxembourg Institute of Health, Luxembourg; laurent.malisoux@lih.lu; Luxembourg Institute of Health, Luxembourg; laurent.malisoux@lih.lu

## Abstract

**Purpose:**

This is the first study aiming at describing the volume and pattern of device-measured movement behaviours performed by adults living in Luxembourg, spanning the full intensity spectrum, and including markers of sedentary time accumulation. We also demonstrated the added value of a multidimensional approach for the comparison of sub-populations.

**Methods:**

This observational study included Luxembourg residents aged 18-79y who each provided ≥4 valid days of triaxial accelerometry in 2016-18 (n = 1122). Compliance with the current international physical activity (PA) guideline was quantified. Linear regression models were used to quantify variability in average 24h acceleration (indicative of PA volume), awake-time PA levels, sedentary time and accumulation pattern with sociodemographic characteristics and other covariates. Data were weighted to be nationally representative.

**Results:**

Overall, participants spent 51% of daily sedentary time (12.1 h/day), 11% in light PA (2.7 h/day), 6% in moderate to vigorous PA (MVPA; 1.5 h/day), and remaining time asleep (7.7 h/day). Adherence to the current PA guideline was high (98.1%), but drop to 22% when including only bouts >10min. Although time spent in MVPA did not differ between women and men, average 24h acceleration and light PA were higher in women than men, while men achieved higher average accelerations across the most active periods of the day. Women performed less sedentary time and shorter sedentary bouts. Older participants (≥55y) registered a lower average 24h acceleration and engaged in less MVPA, more sedentary time and longer sedentary bouts. Average 24h acceleration was higher in participants of lower educational attainment, who also performed less sedentary time, shorter bouts, and fewer bouts of prolonged sedentariness. Average 24h acceleration and levels of PA were higher in participants with standing and manual occupations than a sedentary work type, but manual workers registered lower average accelerations across the most active periods of the day. Standing and manual workers accumulated less sedentary time and fewer bouts of prolonged sedentariness than sedentary workers.

**Conclusions:**

Traditional metrics may incompletely reflect differences in the physical behaviour between sub-populations. Breaking up continuous sedentary periods and replacing sedentary time with active alternatives should be the focus of public health initiatives in Luxembourg.

**Funding:**

Luxembourg Institute of Health.

